# Increasing rates of ventilator-associated events: Blame it on COVID-19?

**DOI:** 10.1017/ash.2023.380

**Published:** 2023-09-29

**Authors:** Aditee Dash, Kaila Cooper, Barry Rittmann, Michael Stevens, Gonzalo Bearman, L. Silvia Munoz-Price, Michelle Doll

## Abstract

**Background:** Rates of ventilator-associated events (VAEs), including infection-related ventilator-associated complications (IVACs) and probable ventilator-associated pneumonia (PVAPs) have increased nationwide since the onset of the COVID-19 pandemic. In December 2021, our health system adopted a new electronic medical record (EMR), which changed the way surveillance for VAEs is performed. We reviewed surveillance criteria, COVID-19 status, and culturing practices in attempts to understand why VAE rates continue to be elevated. **Methods:** We collected data on VAE type, culture data, COVID-19 status, and surveillance criteria for all patients meeting NHSN definitions for VAE from 2018 through November 2022. For all patients in 2022 (post-EMR transition), 2 physicians (A.D. and M.D.) manually reviewed documented ventilator settings from flow sheets to validate the automated EMR data, and they evaluated culture data for appropriateness. Cultures were defined as appropriate unless they were included in “pancultures” for leukocytosis without concern for pneumonia documented. Rates were compared using an interrupted time series (ITS) analysis before and after the onset of the COVID-19 pandemic and the EMR transition. Patient level data were compared across periods using the χ^2^ test. All analyses were performed using SAS version 9.4 software. **Results:** COVID-19 has been implicated in the increasing number of VAEs since the pandemic began: 6% of patients in 2020, 18% in 2021, and 23% in 2022 (*P* < .001). The percentage of patients meeting criteria for VAE by positive end-expiratory pressure (PEEP) decreased from 2018 to 2022 (92%, 95%, 93%, 85%, 85%, respectively; *P* = .0004). Patients meeting criteria for VAE by fraction of inspired oxygen (FiO_2_) increased from 2018 to 2022 (9%, 6%, 11%, 17%, 19%, respectively; *P* = .0002). Manual review of 2022 data indicated opportunities for test stewardship in 8 of 65 patients with cultures (12%). ITS analysis revealed that IVAC+ rates were climbing prior to the onset of the COVID-19 pandemic (Fig. 1). We observed a marked increase in rates with the implementation of our new EMR and the changes to our surveillance process (0.32 cases per 100 ventilator days). Manual review of records from 2022 revealed 5 patients in which documentation of ventilator settings to meet VAE diagnosis could not be retrieved from flow sheets. **Conclusions:** COVID-19 continues to affect VAE despite vaccine availability and may partially account for elevated rates nationwide. However, changes in EMR-automated VAE surveillance may also affect rates. Our findings suggest that automated surveillance captures transient or spurious changes in ventilator machine settings that do not accurately represent clinical status. These data may contribute to spurious increases in VAE. More studies are needed to better understand the impact of both COVID-19 and automated surveillance on VAE.

**Figure:**
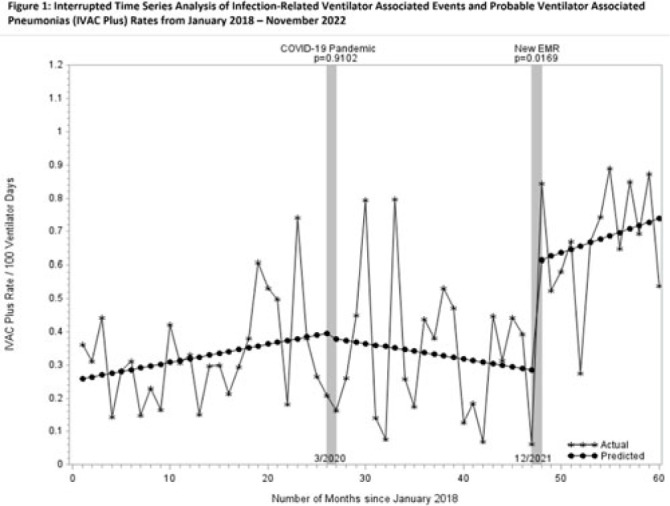

**Disclosures:** None

